# Unilateral Transient Enhanced SEP during Integrated Multiparameter Neurophysiological Monitoring in a Newborn with Symptomatic Seizure

**DOI:** 10.3390/pediatric14020033

**Published:** 2022-05-27

**Authors:** Sara Cavaliere, Silvia Lori, Maria Bastianelli, Cesarina Cossu, Simonetta Gabbanini, Carlo Dani, Giovanna Bertini

**Affiliations:** 1 Department of Neurosciences, Psychology, Drug Research and Children’s Health, University of Florence, 50134 Florence, Italy; sara.cavaliere@unifi.it (S.C.); carlo.dani@unifi.it (C.D.); 2 Neurophysiology Unit, Neuro-Musculo-Skeletal Department, Careggi University Hospital, 50134 Florence, Italy; silvia.lori@unifi.it (S.L.); bastianelli_me@hotmail.com (M.B.); cossuc@aou-careggi.toscana.it (C.C.); simonettagabbanini@virgilio.it (S.G.)

**Keywords:** somatosensory evoked potential, unilateral enhanced SEP, newborn

## Abstract

During Integrated Multiparametric Neurophysiological Monitoring (IMNA), a newborn with suspected hypoxia at birth and microhaemorrhagic and ischaemic lesions presented some clonic-tonic episodes with specific EEG patterns characterized by rolandic and temporal spikes and the appearance of a unilateral enhanced Somatosensory Evoked Potential (SEP) (10.45 µv). Since the literature does not seem to describe cases of giant SEP in newborns, in this case report, we will discuss the hypotheses underlying this potential. It could be assumed that the ischaemic and haemorrhagic lesions presented by the newborn may have developed as a result of neurotransmitter balance failure. This may be the origin of the EEG picture, which, consequently, could have triggered a potential with high amplitude.

## 1. Introduction

The role of somatosensory evoked potential (SEP) combined with EEG in the neonatal setting has already validated its importance in the evaluation of brain damage resulting from haemorrhagic and ischemic insult [1,2,3,4]. The cortical response of the SEPs can be translated as the arrival of the electrical stimulus from the median nerve to the primary somatosensory area. 

The literature describes the normative values of latency and amplitude of this component in adults [5] and children [6]. Our research group has also established normative values for term neonates [1].

The mean amplitude of the cortical response in adults from the median nerve is 0.5–8.7 µV [5], while in neonates (mean gestational age 39.6) it is 2.8–6.5 µV (right) and 2.0–5.5 µV (left) [1].

Regarding the definition of giant SEP, it indicates a cortical potential of significantly increased amplitude. The so-called “giant” somatosensory evoked potentials were first detected by Dawson in 1947 [7], who recorded them in an EEG without averaging techniques [8]. In the literature, the definition of a giant SEP is varied, often unpredictable, and sometimes lacking. Shibasaki et al. judged the SEPs as “giants” when the amplitude, measured from peak-to-peak N20-P25 or P25-N35, was greater than 8.6 µV or 8.4 µV, respectively [9]. Similar values, obtained with the extracephalic reference electrode, were cited by Ebner and Deuschi [10]. Otherwise, Yoshikawa et al. [11] required a minimum of 30 µV, Farnarier et al. [12] a minimum of 40 µV, while Mauguiere et al. instead define giant SEPs as when the N20-P25 cortical component exceeds 15 µV [13]. Considering the imprecise definition of giant SEPs, their diagnosis remains to be defined [8].

The first report describing giant SEPs in patients with myoclonus dates to 1946; Dawson found unusually large SEPs in the precentral area in patients with progressive myoclonic epilepsy [14]. Since that, it has been established that giant SEPs can be registered in the “pyramidal myoclonus” [15] or in the “reflex cortical myoclonus” [16].

The literature provides us with cases of giant SEPs in adults in sepsis, cerebral hypoxia, myoclonus of various origins, progressive supranuclear palsy [17,18], and in some rare conditions such as Rett’s EMS [11], as well as in anaesthesia with etomidate [19]. 

In addition to adults, there are numerous cases of pathologies in children that were characterized by the appearance of this potential. Between 1989 and 1993, 282 children with different neurological diseases were registered as part of the diagnostic studies. In 31 children, the cortical component was greater than in the control group. Four children showed a giant SEP with a value of >40 µV, fifteen showed amplitudes between 20 and 39.9 µV (“high SEP”), and twelve had amplitudes between 14 and 19.9 µV (borderline SEP). There were multiple neurological pathologies of hypoxia at birth or stroke that showed “elevated” SEP [8].

Cases of giant SEPs do not seem to be described in the literature in the neonatal setting. In particular, we do not know the specific biological mechanisms that are at the basis of the onset of this potential.

In this case report, we will examine the case of a full-term newborn with suspected hypoxia at birth, who appears to have an episode of enhanced SEP in conjunction with a seizure.

Since there are no data in the literature that attribute the adjective “giant” to potential with the amplitudes we have recorded, with the same recording setting and age group, we will limit ourselves to defining our potential as “enhanced”, since it is of considerable amplitude versus the mean of the amplitudes obtained by our research group in normative work conducted on newborns [1].

## 2. Case Description

We report the case of a 40-week, 4-day gestational age and 40-week, 5-day postmenstrual age full-term newborn. At birth, the newborn showed an Apgar index of (I) 6 and (V) 9, a funicular pH of 7.03, and a slightly hypotonic appearance.

As the first instrumental examination, an encephalic ultrasound study showed an interhemispheric fissure inclination to the left, and the left lateral ventricle showed the presence of germinal matrix cysts.

Neurological monitoring with aEEG lasting 4 h was performed 30 min after birth, with a normal trace.

**Figure 1:**
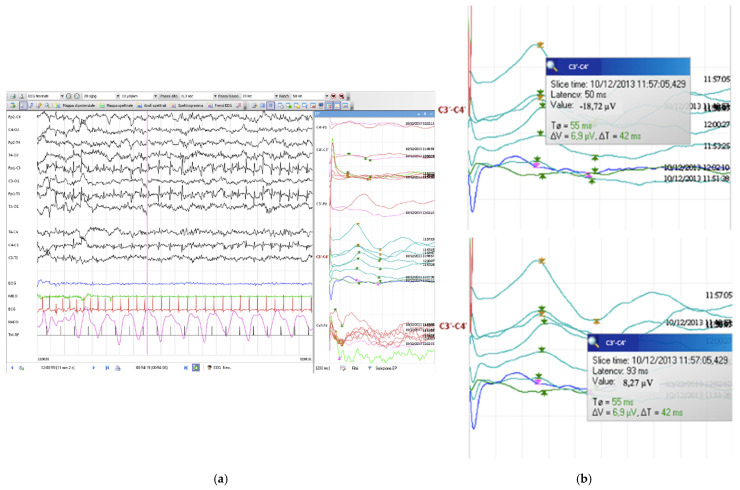
Giant SEPs on the cortical left component during the seizure (**a**), and the measured peak to peak (**b**).

Integrated multiparametric neurophysiological monitoring (IMNA) [1] was clinically indicated. During the monitoring, the newborn presented some episodes characterized by tonic–clonic movements of the left upper limb. These manifestations correlated with specific EEG and SEP patterns, in particular with EEG traces of left rolandic and temporal spikes, grouped in short sequences with a rhythmic aspect, and alternated with short sequences of hypovoltage. In this trace, the cascaded SEPs derived from the left hemisphere were characterized by a significant, sudden, and transient increase in amplitude, 10.45 µV, which seems to coincide with the definition of giant SEPs by Shibasaki et al. [9] (Figure 1). 

**Figure 2:**
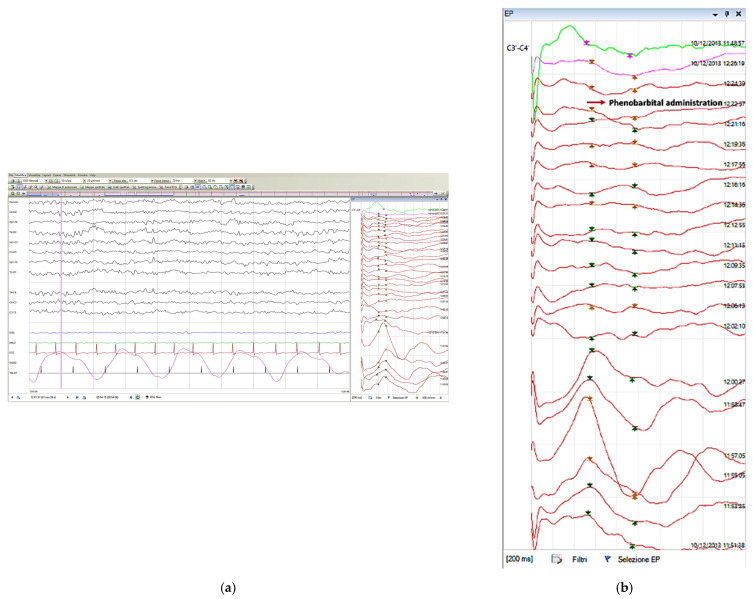
The giant SEP (**a**,**b**) left cortical component after the end of EEG manifestation and phenobarbital administration. (**b**) Chronological order from bottom to top.

Subsequently, with the end of the seizure and following the administration of phenobarbital (1 bolus of 20 mg/kg), the critical anomalies of the EEG traces disappeared. At the same time, the cortical SEPs of the left hemisphere were also normalized in terms of amplitude (3.75 µV) (Figure 2).

An encephalic ultrasound study was repeated after the convulsive episode, which no longer highlighted the inclination of the interhemispheric fissure to the left as the previous one; the symmetry of the cerebral hemispheres was confirmed. The left lateral ventricle with the previously described germinative matrix cyst was more visible.

Other monitoring was then performed: The newborn did not show other similar clinical episodes.

A cranioencephalic MRI study at 7 days of age showed hyperintense spots in left fronto-parietal T1, likely a sign of microhaemorrhages.

In addition, a large area of altered signal appeared at the left parieto-posterior site, extended to the splenium of the corpus callosum, with the restriction of diffusion, compatible with the ischaemic area.

A slight nuance is added in T2, anterior to the left rolandic sulcus and at a deep frontal level. Furthermore, there was bilateral parieto-occipital posterior extracerebral blood collection at the tentorial and retrocerebellar level, which also extended to the vertebral canal.

As a further instrumental examination, the venous and arterial MRI angiography study did not show changes in calibre. The spectroscopic examination performed with the PRESS TE 288 ms technique at the level of the left parietal lesion seemed to be compatible with a picture of subacute ischaemia.

A second cranioencephalic MRI angiography performed as a follow-up at 14 days of life still showed a large area of altered left parietal-posterior signal that affects the corpus callosum to a lesser extent and did not show diffusion restriction, therefore compatible with a picture of chronic ischemia. An initial poromalacic evolution was noted. 

On the other hand, the posterior extracerebral, bilateral parieto-occipital blood collection at the tentorial and retrocerebellar level was reduced, with the extension of the posterior wall of the vertebral canal.

Venous and arterial MRI angiography showed greater congestion of the main vessels, therefore superior sagittal sinus and transverse sinuses.

The last study performed at 30 days of life showed a clear reduction in the blood component, now limited to a minimal presence in the slopes. Outcomes of an ischaemic event were noted at the left posterior parietal level, with enlargement of the subarachnoid spaces. A slight reduction in white matter was also shown. Furthermore, the corpus callosum was limited. Venous and arterial MRI angiographies were normal.

### Neurophysiological Assessments

The newborn was evaluated with simultaneous recording of VEEG and SEPs. According to the small size of the baby’s head, 10 paediatric disposable surface electrodes (including ground and reference electrodes) for EEG recordings were used, in agreement with the International 10–20 System.

To ensure maximum symmetry during electrode positioning, SEPs responses were recorded from cortical and cervical levels using the same electrode as VEEG and the reference electrode at Fz (C3′-Fz, C3′-C4′, C4′-Fz, C4′-C3′, and Cv7-Fz). This double derivation for SEPs cortical responses allows us to identify the cortical component even in cases of dipole variation [20].

Considering that the potential with high amplitude appeared during the recording, we ruled out that it might be due to an asymmetrical positioning of the active electrodes. In this case, the potential would have been asymmetrical from the beginning of the recording. 

## 3. Discussion

We have decided not to define the potential object of our case report as giant since several authors have tried to establish a reference value higher than what the adjective “giant” could be attributed to. However, the literature, to date, does not allow us to find a common parameter setting in reference to the age range of our patient, to concretize the definition of giant SEP. We, therefore, decide to define it as “enhanced”, to mark the considerable increase in amplitude, in the absence of literature confirming a specific terminology in neonates. 

As previously illustrated, the literature to date does not report any cases of giant SEP in newborns, rather only in children. In this case, there are no clinical pictures reported for newborns, as there are for children, that could justify the appearance of giant SEP, such as sepsis, myoclonus, or infantile myoclonic epilepsy [8]. The complete reversibility of the recorded SEP amplitude after the end of the seizure and subsequent phenobarbital administration seems to indicate cortical disinhibition as a probable explanation for both the EEG clinical pattern and related enhanced SEP. It could be deduced that the child’s perinatal hypoxia, together with relative ischaemic and microhaemorrhagic lesions, may have induced the enhanced potential. This reasoning shows similarities with a case described by Schorl Martin et al. in 2008, in which a comatose patient showed the complete regression of the non-convulsive status epilepticus and the giant SEP component following administration of the therapy [21]. 

In fact, pharmacokinetics shows that phenobarbital acts by enhancing the inhibitory action of GABA and reducing the excitatory effects of glutamate [22]. 

From this, by inverse reasoning, this cortical disinhibition can be understood as a temporary loss of GABAergic inhibitions originating at cortical interneurons [23]. This is associated with an undamaged cortical thalamus projection system [24].

The pathophysiology of enhanced/giant cortical SEPs is still uncertain, as well as the nature of the post-excitatory phenomena that likely create them. Neuronal populations that are generators of normal SEPs likely also induce giant N20-P25 and P25-N33 potentials [25], while other components may result from complex neuronal network behaviours and synchronous activation of cortical areas in addition to the primary somatosensory cortex [26].

This is in agreement with what was reported by Valeriani et al. [27], who evaluated giant SEPs in three subjects with cortical myoclonus of unknown cause. They found that the first components had the same tangential dipole found in normal subjects, while the subsequent components showed a different dipole orientation. Furthermore, when mapping the distribution of SEPs components in four patients with PME of various aetiologies, Ikeda et al. [28] highlighted two distinct components of giant SEPs (radial P25–N35 and tangential P30–N30). In the case of EPM1A patients from the study conducted by Visani et al. [26], the presence of a giant N30 suggests the involvement of cortical areas other than the sensory cortex, including the supplementary motor area [29], or sensorimotor interactions.

Furthermore, the rearrangement of the circuits leading to hyperexcitability can sometimes activate “inactive” cortical zones in the derivation of the normal SEP components, thus contributing to the “giant” components [30]. However, the multiplicity of clinical conditions that can associate with giant SEPs makes it difficult to compare the data [26].

This difficulty in finding a common origin or common pathophysiology of these potentials leads us to the likely hypothesis that there is not only a single dipole and a single progression of the event.

Reconstructing the reasoning from the fact that, at the end of the seizure and with the subsequent phenobarbital administration, the newborn showed the disappearance of the EEG and clinical pattern, as well as the correlated enhanced cortical potential, it could be advantageous to consider the ischaemic and haemorrhagic lesion as a factor that may have made the neurotransmitter balance unstable, an equilibrium that a neonatal brain has not yet fully achieved.

The concept necessary to evaluate is the balance of GABA and glutamate in the newborn.

GABA is the main inhibitory neurotransmitter in the brains of older infants, children, and adults, but the activation of chlorine permeable GABA_A_ receptors in foetuses and infants causes excitatory phenomena. The transition from excitatory to inhibitory development in GABA reportedly occurs in the weeks following childbirth, characterized by a decline in NKCC1 expression and an increase in the Cl^−^ - extruding K^+^ -Cl^−^ co-transporter, KCC2 [31].

Glutamate is expressed early in brain development and plays an essential role in activity-dependent plasticity and synaptic refinement [32,33,34,35,36,37].

Both glutamate receptor (NMDA) activation and antagonism are implicated in increasing excitotoxicity and apoptotic neuronal damage, respectively [33,35].

Therefore, perinatal distress or hypoxia can alter these development-regulated transitions and/or can trigger potentially excitotoxic events following perinatal hypoxia/ischaemia that is not detected by conventional neuroimaging [38].

A study conducted by Kilb et al. in 2012 assessed the maturation of GABA from birth to adolescence. During the antenatal period and in the early postnatal phases, GABAergic responses are depolarizing, hence excitatory, and transform into inhibitory responses during the first postnatal weeks [39].

GABAergic depolarization favours dendritic differentiation and the generation of excitatory synapses [40,41], while also facilitating the development of immature oscillatory activity patterns [42].

From the aforementioned study conducted by Sipila et al. in 2005, spontaneous periodic activity emerges as a feature of neuronal network development and is involved in neuronal circuits maturation. In the immature hippocampus, these are defined as “giant depolarizing potentials” (GDPs) during the neuronal development phase, when GABA_A_-mediated transmission is still depolarizing. However, the precise mechanism by which GABAergic transmission promotes the onset of GDP is unknown [42].

## 4. Conclusions

As previously mentioned in the literature, there are no cases of giant SEPs in newborns, and there does not seem to be any evidence of the specific triggering causes.

Considering, therefore, that the glutamate levels increase during perinatal hypoxia; it is not certain that GABA has already developed its inhibitory function during the first days of newborn birth (consequently, there is a lack of neurotransmitter balance); and, lastly, the complete regression of the symptoms (due to ischaemic and microhaemorrhagic lesions) with the end of seizures and the subsequent phenobarbital administration, it can be hypothesized that, in this case, the appearance of an EEG and clinical picture such as the one developed by the newborn and, in parallel, an enhanced SEP, may be the result of these factors. In support of this hypothesis is also the fact that there may be other contributing causes at the basis of this potential, the pathophysiology of which is still not completely known.

## Data Availability

All data are available from the corresponding author upon reasonable requests.
